# Book review

**DOI:** 10.1054/bjoc.2000.1369

**Published:** 2000-09-04

**Authors:** J S Tobias

**Affiliations:** Radiotherapy & Oncology Department of Radiology, Middlesex Hospital, Mortimer Street, London


*British Journal of Cancer* (2000) **83**(7), 971
© 2000 Cancer Research Campaign
doi: 10.1054/ bjoc.2000.1369, available online at http://www.idealibrary.com on IDEAL®

### Book review

### Practical Radiotherapy Planning, 3rd Edn

Jane Dobbs, Ann Barrett and Dan Ash. pp. 394
ISBN 0-340-70631-7, £32.50

This unique radiotherapy planning manual, first published in 1985, remains the most valuable and widely-used practical guide in use today, and goes from strength to strength. Over the years it has evolved from what was initially a handbook of techniques used at the Royal Marsden Hospital, London, to a more widely based volume, the three authors now working at different centres. With this new edition, there's more of a sense of balance, more information, a broader view of the scope of their topic, more flesh on the bones. As before, the bulk of the text covers the many diverse sites of disease with which a clinical oncologist will need to be familiar. There are important preliminary chapters on the essential principles of treatment planning – an excellent guide, by the way, to oncologists of other disciplines who wish to know more about the aims, hazards and limitations of clinical oncology as a technical specialty – as well as updated sections on 3-dimensional radiotherapy planning, conformal therapy, and quality assurance in radiotherapy. The scientific underpinnings of both radiobiology in general, and brachytherapy techniques in particular, are very well covered. The very first illustration of the book, a schematic progression chart describing the relationship between radiotherapy planning, the key preliminary pre-planning phase, and the treatment delivery itself, should be photocopied, laminated, enlarged and displayed in registrars' offices across the country.

As the authors point out in the preface, the book has been rewritten in the new era of evidence-based practice to include rationales and techniques for treatments of proven benefit. To this end, the authors have consulted more widely, though the chief aim remains as before: to provide a guide based on sound anatomical and pathological principles and to cover the practical details of radiotherapy planning, providing advice which is generally unavailable in even the largest oncology texts. For the first time, each chapter includes a few key papers as further reading.

Each of the site-specific sections is well laid out, with brief introductory sections on the role of radiotherapy, the relevant local anatomy, and a balanced and no-nonsense account of the radiotherapy planning techniques, field arrangements and dose prescription. The chapters are well illustrated both by line drawings, CT scans and radiotherapy verification films. I particularly enjoyed the late Professor Tom Wheldon's new chapter on radiobiological principles – an excellent and up-to-date account of current thinking – and the subsequent two chapters on brachytherapy and head and neck radiotherapy planning. All in all, an invaluable guide.

*Dr J S Tobias MD FRCP FRCR*
*Consultant in Radiotherapy & Oncology*
*Department of Radiology*
*Middlesex Hospital*
*Mortimer Street, London*

